# Peak particle velocity data acquisition for monitoring blast induced earthquakes in quarry sites

**DOI:** 10.1016/j.dib.2018.04.103

**Published:** 2018-05-04

**Authors:** O.S. Hammed, O.I. Popoola, A.A. Adetoyinbo, M.O. Awoyemi, T.A. Adagunodo, O. Olubosede, A.K. Bello

**Affiliations:** a Department of Physics, Federal University, Oye-Ekiti, Nigeria; b Department of Physics, University of Ibadan, Nigeria; c Department of Physics, Obafemi Awolowo University, Ile-Ife, Nigeria; d Department of Physics, Covenant University, Ota, Nigeria; e Department of Physical Sciences, Bells University of Technology, Ota, Nigeria

**Keywords:** Quarry blasts, Ground vibration, Peak particle velocity data, Blast seismograph

## Abstract

The peak particle velocity datasets recorded during quarry blasts in the neighborhood villages and towns in Ibadan and Abeokuta were processed and analyzed in order to recommend a safe blast design for each of the quarries. The minimum peak particle velocity of 48.27 mm/s was recorded near the foundation of the nearest residence at the shot to monitored distance of 500 m. The tendency of ground vibration emanating from the quarry sites to cause damage to the structures in the nearby dwelling areas is very high. The peak particle velocity datasets recorded were not within the safe limit. Therefore the peak particle velocity that will not exceed 35 mm/s is recommended for a safe blast design.

**Specifications Table**

**Table:** 

Subject area	Physics
More specific subject area	Solid Earth Physics
Type of data	Tabular text files
How data was acquired	Field surveys using GPS, Blast seismograph (V9000 Seismograph) manufactured by Vibrock Limited,
Data format	Processed and analyzed
Experimental factors	N/A
Experimental features	Processing of raw data into the tabular text files and linear regression lines with brief description of the experiment
Data source location	Field surveys were conducted at five major quarry sites each in Ibadan and Abeokuta Areas, Nigeria. Ibadan Quarry Sites are: Ladson, 7.37°N,3.97°E; Offa, 7.38°N, 3.95°E; Seedvest,7.32°N, 3.92°E; Wetipp ,7.35°N, 3.87°E and Ratcon, 7.33°N,3.87°E. Abeokuta Quarry Sites are: Equation, 7.08°N,3.67°E; Verytaces, 7.15°N, 3.74°E; Phoenix, 7.18°N,3.73°E; Associated, 7.05°N,3.33°E and United, 7.06°N, 3.33°E.
Data accessibility	Data are available within this article

**Value of the data**

• The data could be used as a source of information for quarry blasters to determine the relationship between the peak particle velocity and ground vibration.
• The data could be used to monitor the level of damage on structures in the neighborhood of the quarry sites.
• The data revealed Shot - Monitored distances (the distance between the shot points at the quarry sites and Building Monitored Station Points in the neighborhood of quarry sites which is very useful to quarry blasters to ensure the safety of lives and properties of residents.
• The data could serve as a measuring indicator that can be used by the Environmental protection Agency to ensure that the Peak Particle Velocity values do no exceed 50.8 mm/s recommended by the United State Bureau of Mine.

## 1 Data

The datasets presented here were recorded, using blast seismograph, during the blast induced earthquake triggered at ten quarry sites in Ibadan and Abeokuta areas, Nigeria. The data comprise peak particle velocity (longitudinal, vertical and transverse components), shot-monitored distance, charge weight and scaled distance. Of all the parameters in the datasets, the peak particle velocity dataset is the blast induced earthquake predictor. Blair [1] indicated that the peak particle velocity was strongly dependent upon the maximum charge weight in the near field and the total charge in the very far-field. On the contrary, Singh et al. [2], Singh and Vogt [3] stated that the charge weight could affect the ground vibration only at distances close to the blasts with the effects that diminish quickly with distance. And ultimately it is the charge weight that controls the ground vibration. Olofsson [4] and Persson [5] stated that the magnitude of ground vibrations depended on the quantity of explosives, characteristics of the rock, distance from the blasting site and geology of the deposits.

Pal Roy [6] carried out field investigations with blasting having charge weight varying from 0.5 to 220 t to study the influence of blast duration on ground vibrations. The results signified Charge weight (quantity of explosives) distinctively as the parameter responsible for the persistence of ground movement. However, Zhang [7] observed that the duration of the seismic waves is directly proportional to the charge weight in a blast and inversely proportional to the distance.

The components of these datasets were considered to minimize the complaints of the residents in the neighborhood of the quarry sites. In recent years, one of the problems encountered by technical personnel who are responsible for excavation with blasting is the rightful or unjustifiable complaints of people or organizations in the neighborhood of quarry sites [8], [9]. The number of these kinds of real or psychological disturbances has gradually increased with the increase in the population and urbanization. Therefore, an economical and safe blasting data should eliminate these kinds of problems at the same time. For this reason, one of the significant aspects of good blasting is safety in terms of environmental effects. One of the requirements to be met by blasting design is to determine the maximum amount of explosive per delay for a certain distance, especially in large blasts, and to be able to perform controlled blasting for the elimination of these environmental problems [10], [11]. Experimental studies by explosive producers and users are being continued to determine the effects of ground vibrations and air blast induced by blasting and to be able to take necessary precautions. Legal regulations related to this subject are being developed [12], [13], [14]. Rock blasting in urban areas creates annoying ground vibrations and also may inflict structural damage when excess quantity of explosives is used. Only about 20–30% of the energy expended, during the blasting, is utilized to fragment the rock [15]. Rock breakage process continues after the fracturing of the rock is initiated until the useful energy level becomes less than the strength of the rock [16], [17]. The seismic energy released after the explosion creates a rock deformation through the particle motion. This particle motion generates ground vibrations [18], [19]. The vibration problem may be minimized through the use of proper blast design techniques. The intensity of ground vibration is influenced by a number of factors such as the quantity of explosives, distance from the blast, blast geometry, etc. [20], [21]. Peak particle velocity (PPV) value which is the key for the damage level prediction has been used to develop specification in several countries [22]. Particle velocity is the primary concept for investigating the damage potential of blast-induced vibrations [3].

## 2 Experimental design, materials and methods

The data were generated during the survey of residential buildings in the neighborhood of the quarry sites. A Global Positioning System (GPS) was used to measure Shot-Monitored distances (the distance between the shot points at the quarry sites and Building Monitored Station Points (BMSP) in the neighbourhood of quarry sites). Shot-Monitored distances (D) were recorded from GPS. 3-component blast Seismographs were positioned at twenty BMSP in the dwelling areas surrounding each site. Longitudinal, vertical and transverse PPV datasets associated with the blast-induced earthquakes were recorded from the Seismograph.

Twenty PPV datasets were obtained for each site (as shown in Table 1, Table 2, Table 3, Table 4, Table 5, Table 6, Table 7, Table 8, Table 9, Table 10).

**Table 1:** Datasets of ground vibration measurements and design parameters for blasting at Ladson Quarry.

Data monitoring points	Shot to monitored distance (D) [metres]	Charge weight (in 50 holes) (W) [kg]	Peak Particle velocity (mm/s)				Scaled distance $D/W^{\frac{1}{2}}$
			Vertical [mm/s]	Longitudinal [mm/s]	Transversal [mm/s]	Vectoral Sum [mm/s]	
1	300	1850	110.50	109.45	108.20	189.46	6.97
2	350	1850	86.25	85.95	86.15	149.16	8.14
3	400	1875	70.45	71.25	69.65	122.17	9.24
4	450	1950	60.20	60.35	60.10	104.30	10.20
5	500	1950	50.90	50.20	49.75	87.10	11.32
6	550	1950	43.70	43.85	43.15	75.46	12.46
7	600	1950	39.50	40.50	38.95	68.68	13.59
8	650	1875	32.35	32.75	32.45	56.32	15.01
9	700	1875	28.80	27.50	28.10	48.74	16.17
10	750	1875	25.75	24.95	24.85	43.62	17.32
11	800	1912.5	23.60	22.65	23.85	40.48	18.29
12	850	1912.5	21.45	21.15	20.95	36.69	19.44
13	900	1912.5	19.55	19.15	19.85	33.81	20.58
14	950	1900	17.85	18.95	17.25	31.23	21.79
15	1000	1875	16.25	16.85	15.75	28.21	23.09
16	1050	2950	21.65	20.75	21.15	36.70	19.33
17	1100	1800	13.50	13.05	13.25	22.98	25.93
18	1150	2750	17.65	18.75	17.25	30.99	21.93
19	1200	2150	13.55	13.05	13.95	23.42	25.88
20	1250	2050	12.20	11.75	12.55	21.08	27.61

**Table 2:** Datasets of ground vibration measurements and design parameters for blasting at Offa Quarry.

Data monitoring points	Shot to monitored distance (D) [metre]	Charge weight (in 50 holes) (W) [kg]	Peak Particle velocity (mm/s)				Scaled distance $D/W^{\frac{1}{2}}$
			Vertical [mm/s]	Longitudinal [mm/s]	Transversal [mm/s]	Vectoral Sum [mm/s]	
1	300	1450	90.85	90.75	90.25	156.95	7.88
2	350	1450	71.05	71.30	70.95	100.94	9.19
3	400	1400	55.75	55.75	55.08	96.18	10.69
4	450	1450	47.50	47.40	47.35	82.13	11.82
5	500	1400	39.05	39.15	38.95	67.64	13.36
6	550	1500	35.45	35.55	35.75	60.63	14.20
7	600	1200	25.70	25.85	27.45	45.63	17.32
8	650	1850	32.05	32.00	32.05	55.48	15.11
9	700	1850	28.40	28.45	28.35	49.19	16.27
10	750	950	14.95	14.90	14.85	25.81	24.33
11	800	1350	17.80	17.70	17.85	30.80	21.77
12	850	1250	15.25	15.20	15.15	26.33	24.04
13	900	1250	13.95	13.80	12.95	23.51	25.46
14	950	1500	14.75	14.85	14.25	25.32	24.53
15	1000	1500	13.50	13.25	13.35	23.30	25.82
16	1050	1750	14.25	13.95	14.05	24.39	25.10
17	1100	2350	16.75	16.05	16.25	28.32	22.69
18	1150	1350	10.00	9.85	10.05	17.26	31.30
19	1200	1450	9.90	9.85	9.25	16.75	31.51
20	1250	2755	15.50	15.20	14.95	26.36	23.81

**Table 3:** Datasets of ground vibration measurements and design parameters for blasting at Seed Vest Quarry.

Data monitoring points	Shot to monitored distance (D) [metre]	Charge weight (in 50 holes) (W) [kg]	Peak Particle velocity (mm/s)				Scaled distance $D/W^{\frac{1}{2}}$
			Vertical [mm/s]	Longitudinal [mm/s]	Transversal [mm/s]	Vectoral Sum [mm/s]	
1	300	1650	100.75	101.05	99.85	174.16	7.39
2	350	1550	74.90	73.45	73.95	128.35	8.89
3	400	1355	54.30	54.95	53.85	94.17	10.89
4	450	1555	50.25	49.75	50.05	86.63	11.22
5	500	1850	48.75	48.35	48.05	83.80	11.62
6	550	1900	35.95	36.15	36.95	62.24	12.62
7	600	1400	29.15	29.75	28.85	50.67	16.03
8	650	1100	21.15	20.55	20.95	36.17	19.60
9	700	1250	20.80	21.05	20.10	34.77	19.80
10	750	1500	21.55	20.45	21.05	36.41	19.30
11	800	1800	22.45	22.10	21.95	38.42	18.86
12	850	1950	21.75	20.65	20.05	36.85	19.25
13	900	1250	13.90	13.10	13.55	23.42	25.46
14	950	1250	12.75	11.95	12.10	21.25	26.87
15	1000	1200	10.75	11.05	10.45	18.62	28.88
16	1050	1950	15.50	14.55	15.95	26.58	23.78
17	1100	1800	13.05	13.55	12.75	22.73	25.93
18	1150	1800	12.55	11.85	12.25	21.17	27.11
19	1200	1800	11.75	10.65	11.25	19.44	28.28
20	1250	1950	10.25	11.75	10.55	18.83	28.31

**Table 4:** Datasets of ground vibration measurements and design parameters for blasting at Wetipp Quarry.

Data monitoring points	Shot to monitored distance (D) [metres]	Charge weight (in 50 holes) (W) [kg]	Peak Particle velocity (mm/s)				Scaled distance $D/W^{\frac{1}{2}}$
			Vertical [mm/s]	Longitudinal [mm/s]	Transversal [mm/s]	Vectoral Sum [mm/s]	
1	300	1600	110.44	110.42	109.98	191.01	7.50
2	350	1450	82.24	82.26	82.22	142.44	9.19
3	400	1850	80.85	80.81	80.83	140.00	9.30
4	450	1700	64.10	64.15	64.12	111.06	10.91
5	500	1300	45.30	44.95	45.35	78.29	13.87
6	550	1750	48.94	48.85	48.98	84.74	13.15
7	600	1950	46.66	46.65	46.62	80.79	13.59
8	650	1800	39.20	39.25	39.40	68.04	15.32
9	700	1200	26.24	26.15	26.32	45.44	20.21
10	750	1150	23.02	22.95	23.05	39.85	22.12
11	800	1650	27.24	27.15	27.20	47.11	19.69
12	850	1050	17.98	17.85	17.25	30.65	26.23
13	900	1850	24.95	24.55	24.85	42.93	20.92
14	950	1300	17.86	17.65	17.95	28.87	26.35
15	1000	1950	22.25	22.05	22.15	38.37	22.65
16	1050	1100	13.69	13.45	13.65	23.55	31.66
17	1100	1250	14.03	13.85	13.95	24.15	31.11
18	1150	1350	13.91	13.45	13.85	23.80	31.30
19	1200	1500	14.12	13.90	14.05	24.29	30.98
20	1250	1750	14.88	14.80	14.90	25.74	29.88

**Table 5:** Datasets of ground vibration measurements and design parameters for blasting at Ratcon Quarry.

Data monitoring points	Shot to monitored distance (D) [metres]	Charge weight (in 50 holes) (W) [kg]	Peak Particle velocity (mm/s)				Scaled distance $D/W^{\frac{1}{2}}$
			Vertical [mm/s]	Longitudinal [mm/s]	Transversal [mm/s]	Vectoral Sum [mm/s]	
1	300	1500	105.61	104.95	105.24	182.33	7.75
2	350	1950	101.52	101.32	101.55	175.74	7.93
3	400	1850	77.11	77.00	69.88	129.45	9.30
4	450	1100	40.27	40.15	40.33	69.715	13.57
5	500	1050	32.28	31.98	32.17	55.67	15.43
6	550	1750	42.51	42.02	42.45	73.31	13.15
7	600	1200	26.46	26.39	26.42	45.77	17.32
8	650	1400	26.33	26.30	26.35	45.60	17.37
9	700	1700	27.39	27.32	27.43	47.42	16.98
10	750	1350	19.95	19.75	19.84	34.38	20.41
11	800	1800	22.86	22.84	22.79	39.54	18.86
12	850	1300	15.57	15.45	15.52	26.87	23.57
13	900	1550	16.42	16.35	16.40	28.39	22.86
14	950	1200	12.00	12.05	12.02	20.83	27.42
15	1000	1950	16.69	16.66	16.62	28.85	22.65
16	1050	1900	15.00	15.05	15.02	26.02	24.09
17	1100	1100	8.66	8.59	8.62	13.94	33.17
18	1150	1400	8.87	8.89	8.84	15.36	30.74
19	1200	1550	10.01	10.05	10.08	17.40	30.48
20	1250	1650	9.85	9.79	9.81	15.01	30.77

**Table 6:** Datasets of ground vibration measurements and design parameters for blasting at Equation Quarry.

Data monitoring points	Shot to monitored distance (D) [metres]	Charge weight (in 50 holes) (W) [kg]	Peak Particle velocity (mm/s)				Scaled distance $D/W^{\frac{1}{2}}$
			Vertical [mm/s]	Longitudinal [mm/s]	Transversal [mm/s]	Vectoral Sum [mm/s]	
1	300	1400	114.70	114.75	114.72	198.71	8.02
2	350	1250	84.85	84.82	83.97	146.44	9.90
3	400	1600	83.63	82.98	83.45	144.37	10.00
4	450	1150	55.81	55.78	55.84	96.67	13.27
5	500	1000	43.44	43.41	43.52	75.27	15.81
6	550	2050	63.32	63.25	63.30	109.62	12.15
7	600	1350	41.48	41.35	41.44	71.75	16.33
8	650	1250	35.01	34.85	34.96	60.52	18.38
9	700	1650	38.40	38.38	34.42	64.28	17.23
10	750	1250	28.53	28.49	28.51	49.38	21.21
11	800	1900	35.10	35.03	35.06	60.73	18.35
12	850	1350	25.21	25.14	25.26	43.65	23.13
13	900	1300	22.61	22.63	22.58	39.16	24.96
14	950	1700	25.35	25.29	25.31	43.85	23.04
15	1000	1550	22.05	22.12	22.09	38.26	25.40
16	1050	1650	21.51	21.46	21.54	37.24	25.85
17	1100	1500	18.80	18.75	18.72	32.49	28.40
18	1150	1250	15.48	15.39	15.43	26.73	32.53
19	1200	1450	16.20	16.08	16.13	27.95	31.51
20	1250	1750	17.48	17.42	17.51	30.26	29.88

**Table 7:** Datasets of ground vibration measurements and design parameters for blasting at Verytaces Quarry.

Data monitoring points	Shot to monitored distance (D) [metres]	Charge weight (in 50 holes) (W) [kg]	Peak Particle velocity (mm/s)				Scaled distance $D/W^{\frac{1}{2}}$
			Vertical [mm/s]	Longitudinal [mm/s]	Transversal [mm/s]	Vectoral Sum [mm/s]	
1	300	1050	142.91	142.94	142.89	247.53	9.26
2	350	1150	125.71	125.69	125.74	217.742	10.32
3	400	1250	112.80	112.72	112.77	195.31	11.31
4	450	1400	104.95	104.91	103.89	181.15	12.03
5	500	1450	94.62	94.58	94.53	163.81	13.13
6	550	1300	79.28	79.34	79.38	137.41	15.25
7	600	1450	76.31	75.95	76.26	131.94	15.76
8	650	1350	66.56	66.51	66.62	115.29	17.69
9	700	1650	68.65	67.93	68.62	118.47	17.23
10	750	1500	59.83	59.87	59.68	103.57	19.36
11	800	1100	46.17	46.10	46.05	79.86	24.12
12	850	1000	40.63	40.57	40.53	70.28	26.88
13	900	1300	44.34	44.39	44.42	76.87	24.96
14	950	1200	39.68	39.60	39.65	68.66	27.42
15	1000	1700	45.87	45.83	45.78	79.37	24.25
16	1050	1600	41.78	41.66	41.72	72.26	26.25
17	1100	1100	31.71	31.70	31.64	54.88	33.17
18	1150	1050	29.27	29.15	29.21	50.59	35.49
19	1200	1350	32.29	32.35	32.19	55.90	32.66
20	1250	1250	29.40	29.34	29.31	50.84	35.36

**Table 8:** Datasets of ground vibration measurements and design parameters for blasting at Phoenix Quarry.

Data monitoring points	Shot to monitored distance (D) [metres]	Charge weight (in 50 holes) (W) [kg]	Peak Particle velocity (mm/s)				Scaled distance $D/W^{\frac{1}{2}}$
			Vertical [mm/s]	Longitudinal [mm/s]	Transversal [mm/s]	Vectoral Sum [mm/s]	
1	300	1550	79.83	79.80	79.88	138.28	7.62
2	350	1600	61.13	61.22	61.18	105.96	8.75
3	400	1650	48.67	48.53	48.62	84.19	9.85
4	450	1450	34.23	34.15	34.20	59.22	11.82
5	500	1950	37.17	37.32	37.24	64.51	11.32
6	550	1750	27.86	27.91	27.83	48.27	13.15
7	600	1550	20.95	20.87	20.90	36.21	15.24
8	650	1250	14.59	14.50	14.63	25.24	18.38
9	700	1350	13.62	13.57	13.51	23.50	19.05
10	750	1450	12.77	12.70	12.82	22.11	19.70
11	800	1450	11.27	11.22	11.19	19.45	21.01
12	850	1500	10.36	10.42	10.39	18.00	21.95
13	900	1600	9.88	9.75	9.82	17.00	22.5
14	950	1350	7.55	7.48	7.61	13.07	25.86
15	1000	1250	6.35	6.27	6.40	10.98	28.28
16	1050	1050	4.89	4.81	4.76	8.35	32.40
17	1100	1100	4.67	4.55	4.63	8.00	33.17
18	1150	1250	4.85	4.80	4.78	8.33	32.53
19	1200	1600	5.67	5.59	5.62	9.75	30.00
20	1250	1750	5.71	5.76	5.82	9.98	29.88

**Table 9:** Datasets of ground vibration measurements and design parameters for blasting at Associated Quarry.

Data monitoring points	Shot to monitored distance (D) [metres]	Charge weight (in 50 holes) (W) [kg]	Peak Particle velocity (mm/s)				Scaled distance $D/W^{\frac{1}{2}}$
			Vertical [mm/s]	Longitudinal [mm/s]	Transversal [mm/s]	Vectoral Sum [mm/s]	
1	300	1200	106.00	106.12	106.04	183.69	8.66
2	350	1150	83.21	83.12	83.19	144.06	10.32
3	400	1800	94.28	94.15	94.22	163.19	9.43
4	450	1850	81.66	81.58	81.52	141.31	10.46
5	500	1950	73.22	73.27	73.31	126.90	11.32
6	550	1450	52.33	52.26	52.20	90.52	14.44
7	600	1350	44.18	44.25	44.09	76.51	16.33
8	650	1450	41.56	41.48	41.61	71.97	17.07
9	700	1500	38.40	38.35	38.46	66.52	18.07
10	750	1750	38.83	38.79	38.88	67.26	17.93
11	800	1650	34.11	33.92	34.19	59.02	19.69
12	850	1700	32.03	32.11	31.98	55.49	20.62
13	900	1750	30.20	30.32	30.27	52.42	21.51
14	950	1600	26.34	26.25	26.30	45.55	23.75
15	1000	1850	27.13	26.85	27.02	46.77	23.25
16	1050	1400	20.93	20.79	20.84	36.12	28.06
17	1100	1450	20.11	20.23	20.16	34.93	28.89
18	1150	1350	18.00	18.19	18.11	31.35	31.30
19	1200	1800	20.70	20.81	20.62	35.87	28.28
20	1250	1950	20.68	20.52	20.60	35.68	28.31

**Table 10:** Datasets of ground vibration measurements and design parameters for blasting at United Quarry.

Data monitoring points	Shot to monitored distance (D) [metres]	Charge weight (in 50 holes) (W) [kg]	Peak Particle velocity (mm/s)				Scaled distance $D/W^{\frac{1}{2}}$
			Vertical [mm/s]	Longitudinal [mm/s]	Transversal [mm/s]	Vectoral Sum [mm/s]	
1	300	850	83.56	83.47	83.66	144.74	10.29
2	350	750	61.96	61.84	61.75	107.13	12.78
3	400	1300	75.32	75.40	74.28	129.91	11.09
4	450	1100	57.05	57.22	57.15	98.97	13.57
5	500	650	34.31	34.38	34.43	59.54	19.61
6	550	1500	53.57	53.48	53.65	92.78	14.20
7	600	1750	52.84	52.72	52.90	91.49	14.34
8	650	950	31.04	31.15	31.24	53.94	21.09
9	700	1000	29.03	29.12	28.86	50.24	22.14
10	750	1300	31.63	31.56	31.72	54.80	20.80
11	800	950	23.31	23.42	23.38	40.48	25.96
12	850	950	21.44	21.56	21.49	37.23	27.58
13	900	1200	23.27	23.08	23.16	40.13	25.98
14	950	1550	25.77	25.62	25.70	44.51	24.13
15	1000	850	15.86	15.92	15.80	27.47	34.30
16	1050	1400	20.93	20.85	20.77	36.11	28.06
17	1100	1400	19.63	19.75	19.81	34.17	29.40
18	1150	700	11.44	11.29	11.38	19.69	43.47
19	1200	1600	19.08	19.22	18.97	33.07	30.00
20	1250	1000	13.04	13.17	13.29	22.81	39.53

The patterns and protocols applied by the quarry blasters during the shots were followed in obtaining these data. For blasting operations at these sites, Ammonium Nitrate Fuel Oil (ANFO) was used as explosive. The explosives were detonated using magnadet detonator. Scaled distance (SD) data were obtained for each BMSP (as shown in Table 1, Table 2, Table 3, Table 4, Table 5, Table 6, Table 7, Table 8, Table 9, Table 10) using:

(1) $$SD=D\times W^{-0.5}$$

Charge weight of explosive, W, tends to W_m_, when the quantity of explosive is maximum.

**Fig. 1:**
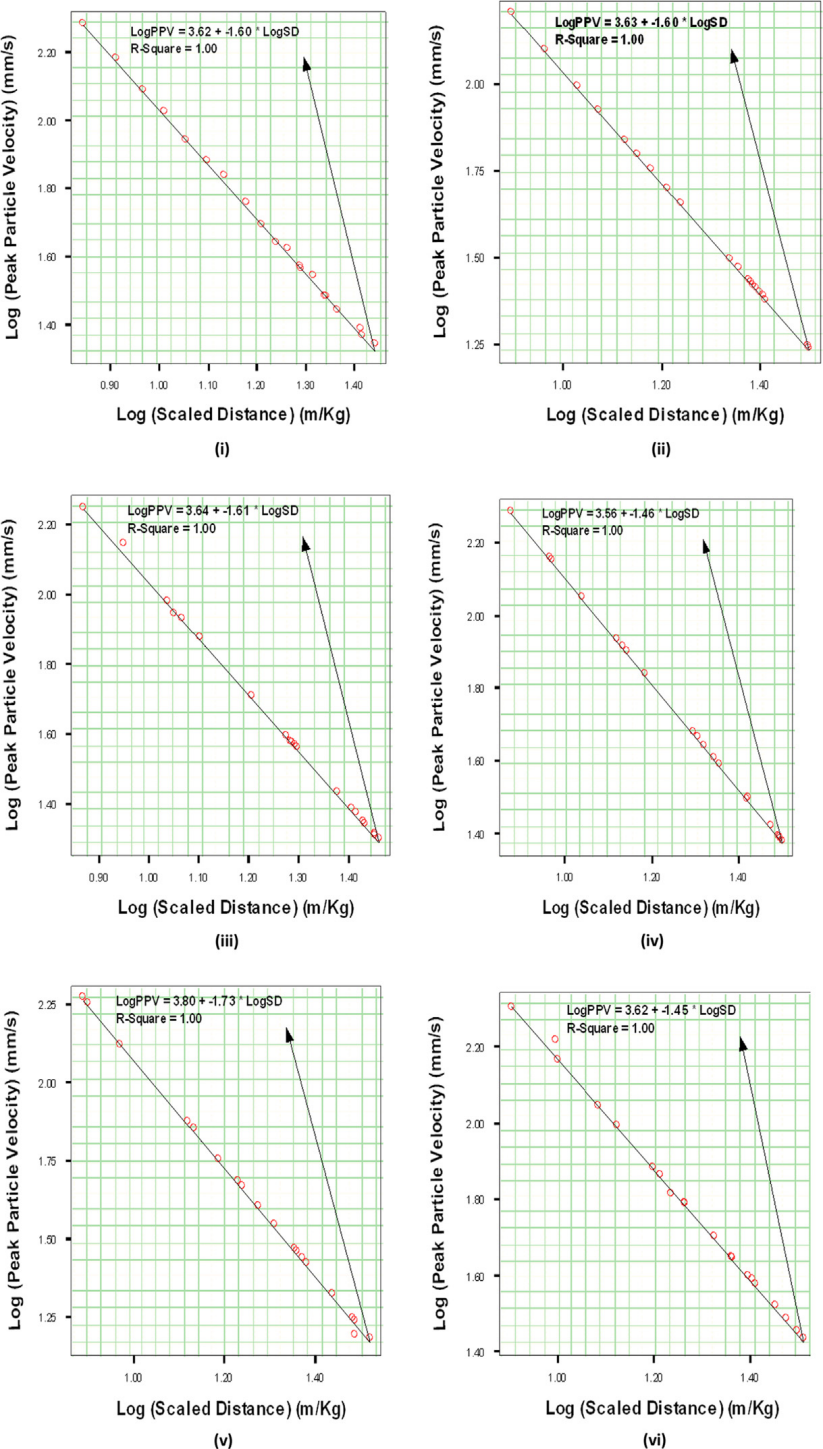

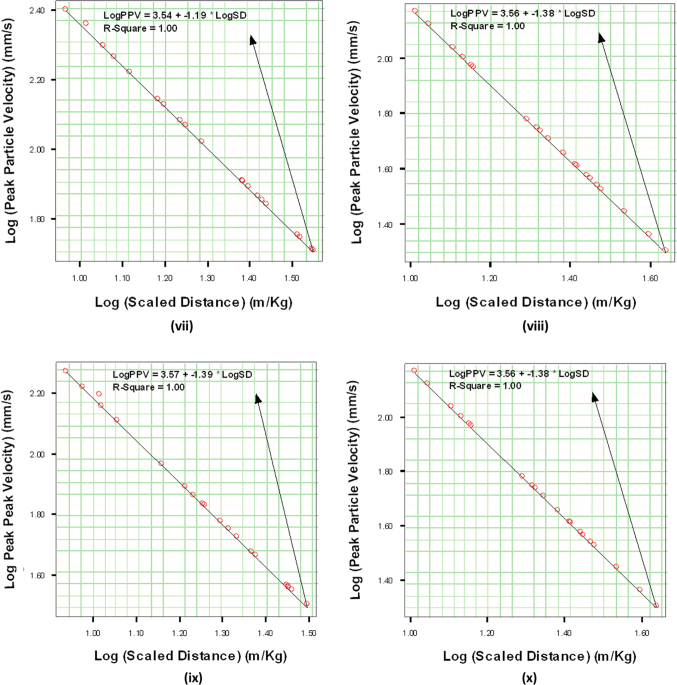
Logarithmic plot of Peak Particle Velocity against Scaled Distance datasets at Five major Quarry Sites each in Ibadan and Abeokuta, Nigeria. Ibadan quarry sites are: (i) Ladson (ii) Offa (iii) Seedvest (iv) Wetipp and (v) Ratcon. Abeokuta quarry sites are: (vi) Equation, (vii) Verytaces, (viii) Phoenix, (ix) Associated and (x) United.

In order to establish a useful relationship between PPV and SD, as shown in Eq. (2), the PPV against SD data pairs obtained for each site was plotted using linear regression lines (Fig. 1).

(2) logPPV=logK−βlogSD

K and β are rock energy transfer and attenuation constants of the sites determined from the log PPV versus log SD linear regression graphs.

The maximum quantity of explosives (W_m_) that can be detonated safely was determined using Eqs. (1), (2).

The PPV datasets obtained from ground vibrations at all the monitored stations recorded ranged from 9.98 to 247.53 mm/s. The PPV exceeded 50.8 mm/s recommended by the United States Bureau of Mine (USBM) at 55% of BMSP. The PPV data revealed that the vibration intensities were not bearable at most of the monitored stations. The analysis of data signifies that there is a correlation between the exceeded or large PPV recorded and the cracked walls of buildings in the vicinity of quarry sites.

A combination of scaled distance and geological constants, k and β parameters obtained from the regression lines in Fig. 1, could be used to predict the exact PPV associated with the vibration intensities of the quarry sites. Combination of these parameters with the PPV could also be used to determine the maximum quantity of the explosive to be detonated that will not cause damage to the buildings and structures around the sites.
