# Supplementary material for: Peak particle velocity data acquisition for monitoring blast induced earthquakes in quarry sites
Source: Data Brief. 2018 May 4;19:398–408. doi: 10.1016/j.dib.2018.04.103 (PMC5997587; doi:10.1016/j.dib.2018.04.103)
Supplement: Supplementary file 1 — Supplementary material [file mmc1.doc]

There is no conflict of interest as far as this work is concerned.
